# Crystal structure, Hirshfeld surface analysis and DFT studies of ethyl 2-{4-[(2-eth­oxy-2-oxoeth­yl)(phen­yl)carbamo­yl]-2-oxo-1,2-di­hydro­quinolin-1-yl}acetate

**DOI:** 10.1107/S2056989019014154

**Published:** 2019-10-29

**Authors:** Yassir Filali Baba, Sonia Hayani, Tuncer Hökelek, Manpreet Kaur, Jerry Jasinski, Nada Kheira Sebbar, Youssef Kandri Rodi

**Affiliations:** a Laboratoire de Chimie Organique Appliquée, Université Sidi Mohamed Ben Abdallah, Faculté des Sciences et Techniques, Route d’immouzzer, BP 2202, Fez, Morocco; b Department of Physics, Hacettepe University, 06800 Beytepe, Ankara, Turkey; c Department of Chemistry, Keene State College, 229 Main Street, Keene, NH 03435-2001, USA; d Laboratoire de Chimie bioorganique appliquée, Faculté des sciences, Université Ibn Zohr, Agadir, Morocco; e Laboratoire de Chimie Organique Hétérocyclique URAC 21, Pôle de Compétence Pharmacochimie, Av. Ibn Battouta, BP 1014, Faculté des Sciences, Université Mohammed V, Rabat, Morocco

**Keywords:** crystal structure, 2-oxo­quinoline, alkyne, weak inter­molecular inter­actions, π-stacking, Hirshfeld surface

## Abstract

The title com­pound, C_24_H_24_N_2_O_6_, consists of ethyl 2-(1,2,3,4-tetra­hydro-2-oxo­quinolin-1-yl)acetate and 4-[(2-eth­oxy-2-oxoeth­yl)(phen­yl)carbomoyl] units, where the oxo­quinoline unit is almost planar and the acetate substituent is nearly perpendicular to its mean plane. In the crystal, C—H_Oxqn_⋯O_Ethx_ and C—H_Ph­yl_⋯O_Carbx_ (Oxqn = oxoquinolin, Ethx = eth­oxy, Phyl = phenyl and Carbx = carboxyl­ate) weak hydrogen bonds link the mol­ecules into a three-dimensional network sturucture. A π–π inter­action between the constituent rings of the oxo­quinoline unit, with a centroid–centroid distance of 3.675 (1) Å may further stabilize the structure. Both terminal ethyl groups are disordered over two sets of sites. The ratios of the refined occupanies are 0.821 (8):0.179 (8) and 0.651 (18):0.349 (18). The Hirshfeld surface analysis of the crystal structure indicates that the most important contributions for the crystal packing are from H⋯H (53.9%), H⋯O/O⋯H (28.5%) and H⋯C/C⋯H (11.8%) inter­actions. Weak inter­molecular hydrogen-bond inter­actions and van der Waals inter­actions are the dominant inter­actions in the crystal packing. Density functional theory (DFT) geometric optimized structures at the B3LYP/6-311G(d,p) level are com­pared with the experimentally determined mol­ecular structure in the solid state. The HOMO–LUMO mol­ecular orbital behaviour was elucidated to determine the energy gap.

## Abstract 2

The title com­pound, C_24_H_24_N_2_O_6_, consists of ethyl 2-(1,2,3,4-tetra­hydro-2-oxoquinolin-1-yl)acetate and 4-[(2-eth­oxy-2-oxoeth­yl)(phen­yl]carbomoyl units, where the oxo­quinoline unit is almost planar and the acetate substituent is nearly perpendicular to its mean plane. In the crystal, C—H_Oxqn_⋯O_Ethx_ and C—H_Ph­yl_⋯O_Carbx_ (Oxqn = oxoquinolin, Ethx = eth­oxy, Phyl = phenyl and Carbx = carboxyl­ate) weak hydrogen bonds link the mol­ecules into a three-dimensional network structure. A π–π inter­action with a centroid-centroid distance of 3.675 (1) Å between the constituent rings of the oxo­quinoline unit may further stabilize the structure.

## Chemical context   

In recent years, research has been focused on existing mol­ecules and their modifications in order to reduce their side effects and to explore their other pharmacological properties. Quinolone derivatives have constituted an important class of heterocyclic com­pounds which, even when part of a com­plex mol­ecule, possesses a wide spectrum of biological activities, such as anti­cancer (Elderfield & Le Von, 1960 ▸), anti­fungal (Musiol *et al.*, 2010 ▸), anti­tubercular (Fan *et al.*, 2018*a* ▸; Xu *et al.*, 2017 ▸), anti­malarial (Fan *et al.*, 2018*b* ▸; Hu *et al.*, 2017 ▸), anti-HIV (Sekgota *et al.*, 2017 ▸; Luo *et al.*, 2010 ▸), anti-HCV (Manfroni *et al.*, 2014 ▸; Cheng *et al.*, 2016 ▸) and anti­microbial (Musiol *et al.*, 2006 ▸). They have been developed for the treatment of many diseases, like malaria (Lutz *et al.*, 1946 ▸) and HIV (Ahmed *et al.*, 2010 ▸). As a continuation of our research work devoted to the development of N-substituted quinoline derivatives and the assessments of their potential pharmacological activities (Filali Baba *et al.*, 2016*a* ▸, 2017 ▸, 2019 ▸; Bouzian *et al.*, 2018 ▸, 2019*a* ▸), we report herein the synthesis and mol­ecular and crystal structure of the title com­pound, along with the Hirshfeld surface (HS) analysis and density functional theory (DFT) com­putational calculations carried out at the B3LYP/6-311G(d,p) level of an N-substituted quinoline derivative by an alkyl­ation reaction of ethyl bromo­acetate with 2-oxo-*N*-phenyl-1,2-di­hydro­quinoline-4-carboxamide under phase-transfer catalysis conditions using tetra-*n*-butyl­ammonium bromide (TBAB) as a catalyst and potassium carbonate as a base.
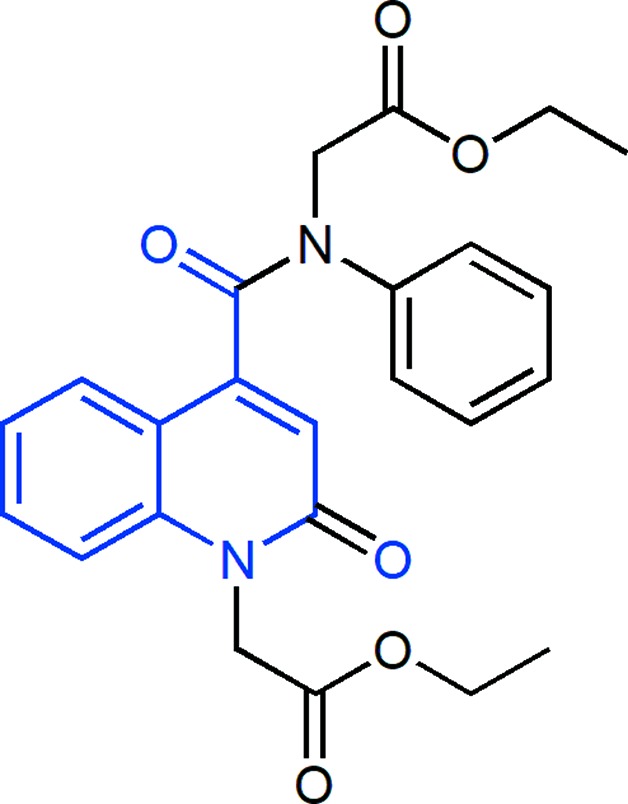


## Structural commentary   

**Figure 1:**
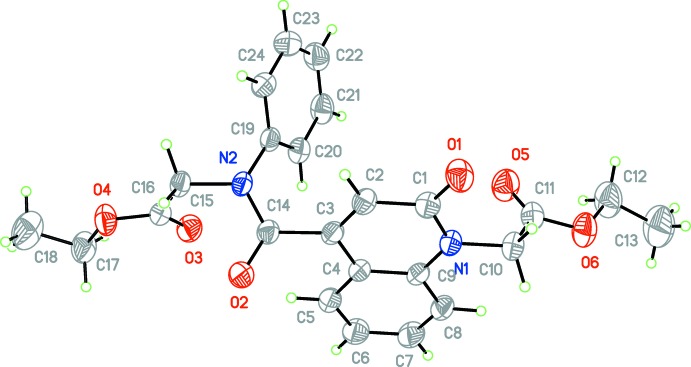
The mol­ecular structure of the title com­pound, showing the atom-numbering scheme and displacement ellipsoids drawn at the 50% probability level. For the sake of clarity, the minor com­ponent of disorder is not shown.

The title mol­ecule is com­posed of ethyl 2-(1,2,3,4-tetra­hydro-2-oxoquinolin-1-yl)acetate and 4-[(2-eth­oxy-2-oxoeth­yl)(phen­yl)carbomo­yl] units (Fig. 1 ▸). The mean planes of the constituent rings, *i.e. A* (atoms N1/C1–C4/C9) and *B* (C4–C9), of the oxo­quinoline unit are oriented at a dihedral angle of 1.04 (6)°. Thus, they are almost coplanar, with a maximum deviation of 0.017 (3) Å for atom C7. Atoms O1 and C10 deviate only by 0.007 (2) and 0.022 (2) Å from that plane and so are essential coplanar. The acetate substituent is nearly perpendicular to that plane, with a torsion angle of C1—N1—C10—C11 = −104.8 (2)°. The mean plane of the phenyl ring, *C* (C19–C24), is oriented with respect to the oxo­quinoline unit at a dihedral angle of 68.17 (6)°. The carboxyl groups, O5/O6/C11 and O3/O4/C16, are twisted out of coplanarity with the best least-squares plane of the oxo­quinoline unit and phenyl ring *C* by dihedral angles of 79.7 (2) and 62.9 (2)°, respectively.

## Supra­molecular features   

**Figure 2:**
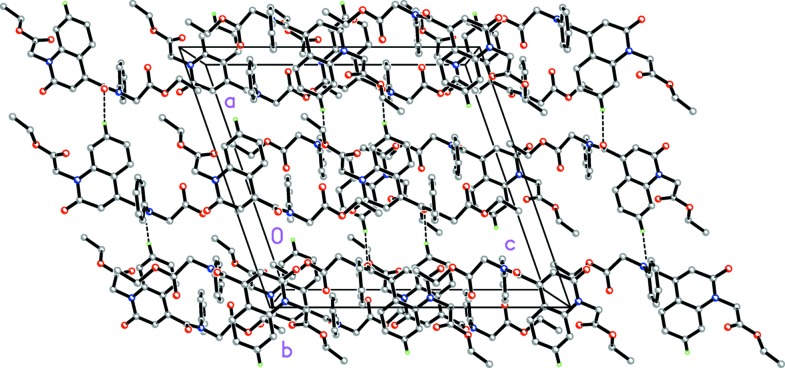
A partial packing diagram viewed along the *b* axis. Weak C—H_Oxqn_⋯O_Ethx_ and C—H_Ph­yl_⋯O_Carbx_ (Oxqn = oxoquinolin, Ethx = eth­oxy, Phyl = phenyl and Carbx = carboxyl­ate) inter­molecular hydrogen bonds are shown as dashed lines. The disorder is not shown.

In the crystal, weak C—H_Oxqn_⋯O_Ethx_ and C—H_Ph­yl_⋯O_Carbx_ (Oxqn = oxoquinolin, Ethx = eth­oxy, Phyl = phenyl and Carbx = carboxyl­ate) hydrogen bonds (Table 1 ▸) link the mol­ecules into a three-dimensional network structure (Fig. 2 ▸). A π–π contact between the constituent rings, *i.e.* *A* (N1/C1–C4/C9) and *B* (C4–C9), of the oxo­quinoline unit, with *Cg*1⋯*Cg*2^i^ = 3.675 (1) Å [symmetry code: (i) −*x* + 1, −*y* + 1, −*z* + 1; *Cg*1 and *Cg*2 are the centroids of rings *A* and *B*], may further stabilize the structure. The Hirshfeld surface analysis of the crystal structure indicates that the most important contributions for crystal packing are from H⋯H (53.9%), H⋯O/O⋯H (28.5%) and H⋯C/C⋯H (11.8%) inter­actions. Weak inter­molecular hydrogen-bond inter­actions and van der Waals inter­actions are the dominant inter­actions in the crystal packing.

## Hirshfeld surface analysis   

**Figure 3:**
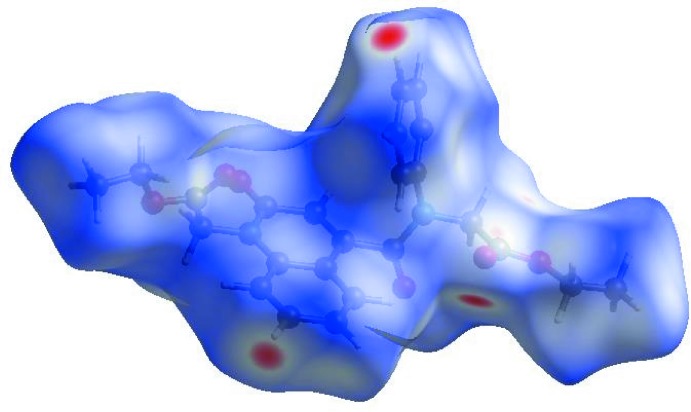
A view of the three-dimensional Hirshfeld surface for the title com­pound, plotted over *d*_norm_ in the range −0.2380 to 1.5740 a.u.

**Figure 4:**
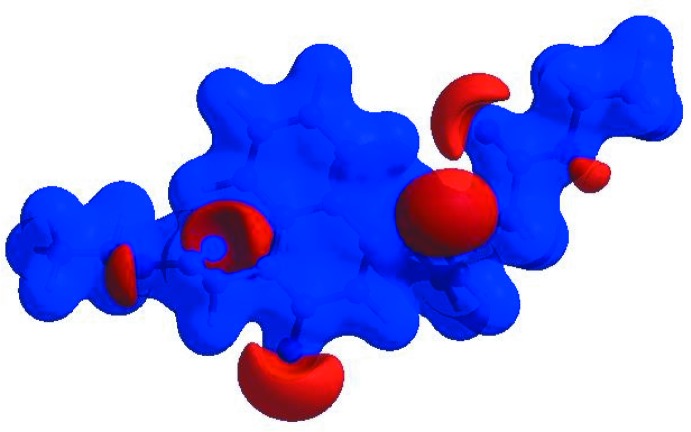
A view of the three-dimensional Hirshfeld surface of the title com­pound, plotted over the electrostatic potential energy in the range −0.0500 to 0.0500 a.u. using the STO-3G basis set at the Hartree–Fock level of theory. Weak hydrogen-bond donor and acceptor inter­molecular inter­actions are shown as blue and red regions around the atoms corresponding to positive and negative potentials, respectively.

**Figure 5:**
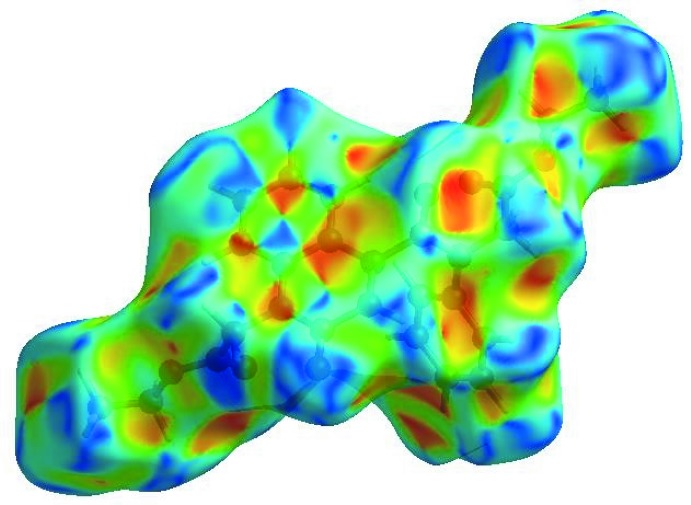
A view of the Hirshfeld surface for the title com­pound, plotted over the shape-index.

**Figure 6:**
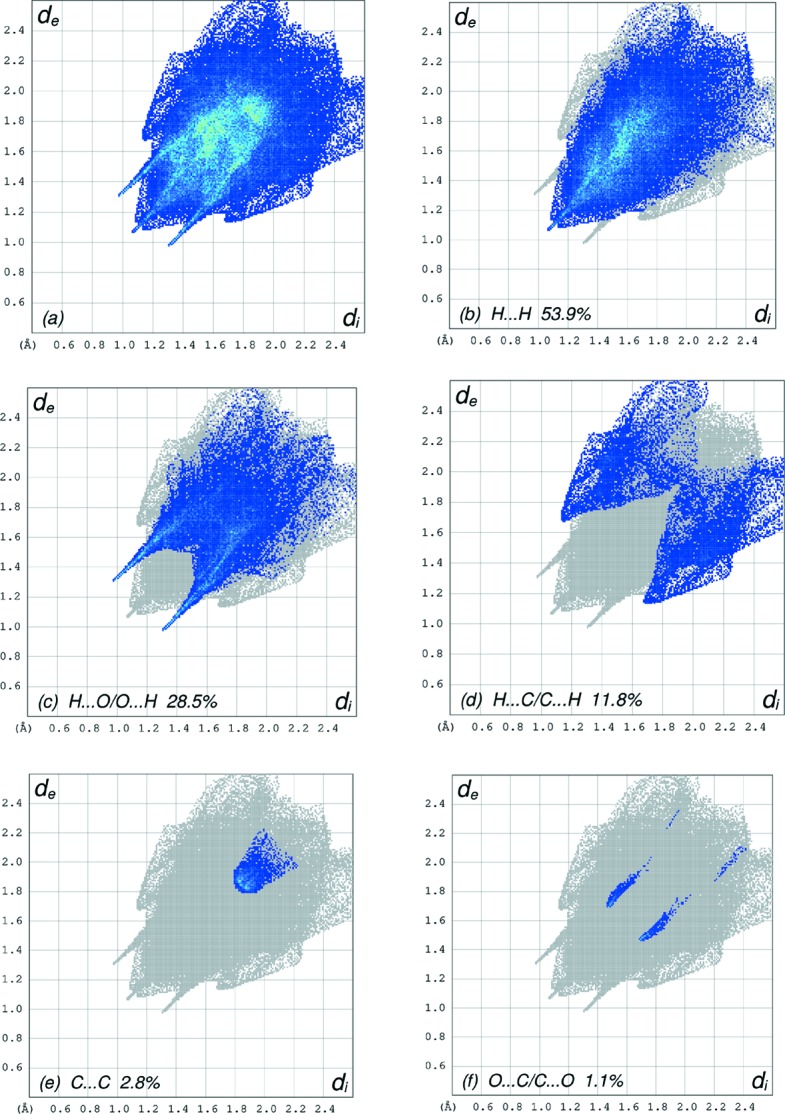
The full two-dimensional fingerprint plots for the title com­pound, showing (*a*) all inter­actions, and delineated into (*b*) H⋯H, (*c*) H⋯O/O⋯H, (*d*) H⋯C/C⋯H, (*e*) C⋯C and (*f*) O⋯C/C⋯O inter­actions. The *d*_i_ and *d*_e_ values are the closest inter­nal and external distances (in Å from given points on the Hirshfeld surface contacts.

In order to visualize the inter­molecular inter­actions in the title com­pound, a Hirshfeld surface (HS) analysis (Hirshfeld, 1977 ▸; Spackman & Jayatilaka, 2009 ▸) was carried out by using *CrystalExplorer17.5* (Turner *et al.*, 2017 ▸). In the HS plotted over *d*_norm_ (Fig. 3 ▸), the white surface indicates contacts with distances equal to the sum of the van der Waals radii, and the red and blue colours indicate distances shorter (in close contact) or longer (distinct contact) than the van der Waals radii, respectively (Venkatesan *et al.*, 2016 ▸). The bright-red spots appearing near O2 and H atoms H7 and H22 indicate their roles as the respective donors and/or acceptors; they also appear as blue and red regions corresponding to positive and negative potentials on the HS mapped over the electrostatic potential (Spackman *et al.*, 2008 ▸; Jayatilaka *et al.*, 2005 ▸), as shown in Fig. 4 ▸. The blue regions indicate a positive electrostatic potential (hydrogen-bond donors), while the red regions indicate a negative electrostatic potential (hydrogen-bond acceptors). The shape-index of the HS is a tool to visualize the π–π stacking by the presence of adjacent red and blue triangles; if there are no adjacent red and/or blue triangles, then there are no π–π inter­actions. Fig. 5 ▸ clearly suggests that there are π–π inter­actions in (I). The overall two-dimensional fingerprint plot (Fig. 6 ▸ *a*) and those delineated into H⋯H, H⋯O/O⋯H, H⋯C/C⋯H, C⋯C and O⋯C/C⋯O contacts (McKinnon *et al.*, 2007 ▸) are illustrated in Figs. 6 ▸(*b*)–(*f*), respectively, together with their relative contributions to the Hirshfeld surface. The most important inter­action is H⋯H, contributing 53.9% to the overall crystal packing, which is reflected in Fig. 6 ▸(*b*) as widely scattered points of high density due to the large hydrogen content of the mol­ecule, with the tip at *d*_e_ = *d*_i_ = 1.05 Å, due to the short inter­atomic H⋯H contacts. The pair of characteristic wings resulting in the fingerprint plot delineated into H⋯O/O⋯H contacts (Fig. 6 ▸ *c*) has a 28.5% contribution to the HS and is viewed as a pair of spikes with the tips at *d*_e_ + *d*_i_ = 2.30 Å. In the absence of weak C—H⋯π inter­actions, the pair of characteristic wings resulting in the fingerprint plot delineated into H⋯C/C⋯H contacts (Fig. 6 ▸ *d*), with a 11.8% contribution to the HS and are viewed as a pair of spikes with the tip at *d*_e_ + *d*_i_ = 2.83 Å. The C⋯C contacts (Fig. 6 ▸ *e*) have an arrow-shaped distribution of points with the tip at *d*_e_ = *d*_i_ = 1.81 Å. Finally, the pair of the scattered points of wings from the fingerprint plot are delineated into O⋯C/C⋯O (Fig. 6 ▸ *f*) contacts, with a 1.1% contribution to the HS, and has a nearly symmetrical distribution of points with the edges at *d*_e_ + *d*_i_ = 3.15 Å.

**Figure 7:**
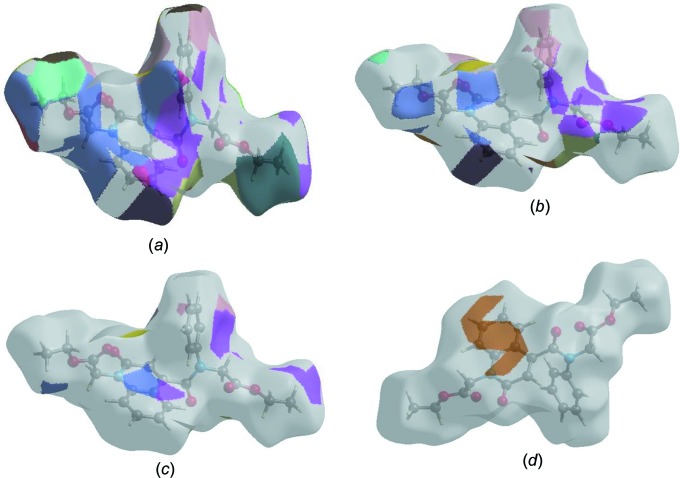
The Hirshfeld surface representations with the function *d*_norm_ plotted onto the surface for (*a*) H⋯H, (*b*) H⋯O/O⋯H, (*c*) H⋯C/C⋯H and (*d*) C⋯C inter­actions.

The Hirshfeld surface representations with the function *d*_norm_ plotted onto the surface are shown for the H⋯H, H⋯O/O⋯H, H⋯C/C⋯H and C⋯C inter­actions in Figs. 7 ▸(*a*)–(*d*), respectively.

The Hirshfeld surface analysis confirms the importance of weak H-atom contacts in establishing the packing structure. The large number of H⋯H, H⋯O/O⋯H and H⋯C/C⋯H inter­actions suggest that van der Waals inter­actions and weak hydrogen-bond inter­molecular inter­actions play major roles in the crystal packing (Hathwar *et al.*, 2015 ▸).

## DFT calculations   

**Figure 8:**
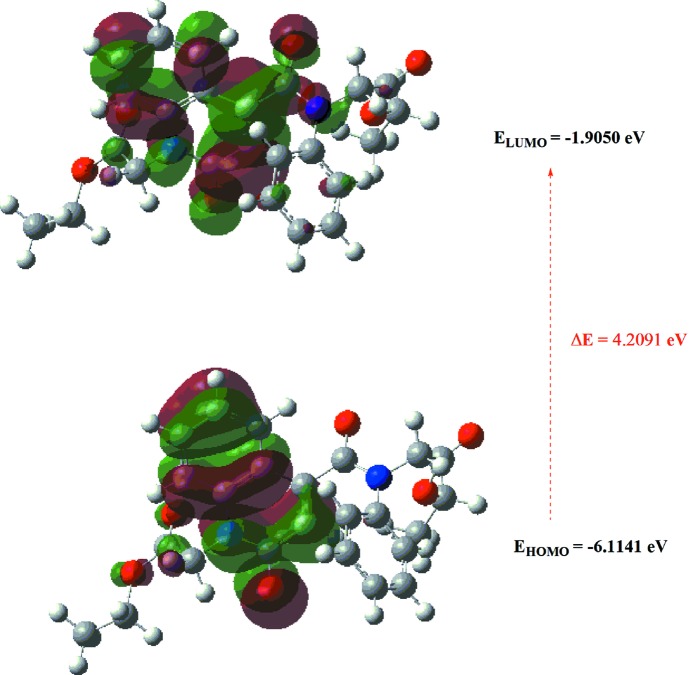
The calculated energy band gap for the title com­pound.

The geometry optimized structure of the title com­pound in the gas phase was generated theoretically *via* density functional theory (DFT) com­putational calculations using a standard B3LYP functional and a 6-311G(d,p) basis set (Becke, 1993 ▸), as implemented in *GAUSSIAN09* (Frisch *et al.*, 2009 ▸). The theoretical and experimental results were in good agreement (Table 2 ▸). A DFT mol­ecular orbital calculation indicated that the highest-occupied mol­ecular orbital (HOMO), acting as an electron donor, and the lowest-unoccupied mol­ecular orbital (LUMO), acting as an electron acceptor, are very important parameters for quantum chemistry. When the energy gap is small, the mol­ecule is highly polarizable and has high chemical reactivity. Therefore, these DFT calculations provide important information on the reactivity and site selectivity of the mol­ecular framework. *E*_HOMO_ and *E*_LUMO_ clarify the inevitable charge exchange collaboration inside the studied material, as well as electronegativity (χ), hardness (η), potential (μ), electrophilicity (ω) and softness (σ), which are listed in Table 3 ▸. The significance of η and σ is to evaluate both reactivity and stability. The electron transition from a HOMO to a LUMO energy level is shown in Fig. 8 ▸. The HOMO and LUMO are localized in the plane extending from the whole ethyl 2-{4-[(2-eth­oxy-2-oxoeth­yl)(phen­yl)carbamo­yl]-2-oxo-1,2-di­hydro­quinolin-1-yl}acetate ring. The energy band gap [Δ*E* = *E*_LUMO_ − *E*_HOMO_] of the mol­ecule was about 4.2091 eV, and the frontier mol­ecular orbital (FMO) energies, *i.e. E*_HOMO_ and *E*_LUMO_, were −6.1141 and −1.9050 eV, respectively.

## Database survey   

A non-a­lkylated analogue, namely quinoline and its derivatives, has been reported (Filali Baba *et al.*, 2016*b* ▸, 2017 ▸; Bouzian *et al.*, 2019*a* ▸), as well as three similar structures (see Castañeda *et al.*, 2014 ▸; Kafka *et al.*, 2012 ▸; Bouzian *et al.*, 2018 ▸, 2019*a* ▸; Divya Bharathi *et al.*, 2015 ▸).

## Synthesis and crystallization   

To a solution of 2-oxo-*N*-phenyl-1,2-di­hydro­quinoline-4-carboxamide (1.89 mmol) in di­methyl­formamide (DMF, 10 ml) were added ethyl bromo­acetate (4.16 mmol), K_2_CO_3_ (5.67 mmol) and tetra­butyl­ammonium bromide (TBAB, 0.23 mmol). The reaction mixture was stirred at room temperature for 6 h. After removal of the salts by filtration, the DMF was evaporated under reduced pressure and the resulting residue was dissolved in di­chloro­methane. The organic phase was dried with Na_2_SO_4_ and then concentrated under reduced pressure. The pure com­pound was obtained by column chromatography using as eluate hexa­ne/ethyl acetate (3:1 *v*/*v*). The isolated solid was recrystallized from hexa­ne–diethyl acetate (1:1 *v*/*v*) to afford colourless crystals (yield 75%; m.p. 427 K).

## Refinement   

The experimental details, including the crystal data, data collection and refinement, are summarized in Table 4 ▸. H atoms were positioned geometrically, with C—H = 0.93, 0.97 and 0.96 Å for aromatic CH, CH_2_ and CH_3_ H atoms, respectively, and constrained to ride on their parent atoms, with *U*_iso_(H) = *kU*_eq_(C), where *k* = 1.5 for CH_3_ H atoms and *k* = 1.2 for other H atoms. The terminal ethyl groups are disordered with an occupancy ratio of 0.821 (8):0.179 (8) for C12 and C13, and 0.651 (18):0.349 (18) for C17 and C18.

## Supplementary Material

Crystal structure: contains datablock(s) I. DOI: 10.1107/S2056989019014154/lh5924sup1.cif

Structure factors: contains datablock(s) I. DOI: 10.1107/S2056989019014154/lh5924Isup2.hkl

CCDC references: 1959642, 1959642

Additional supporting information:  crystallographic information; 3D view; checkCIF report

## Figures and Tables

**Table 1:** Hydrogen-bond geometry (Å, °)

*D*—H⋯*A*	*D*—H	H⋯*A*	*D*⋯*A*	*D*—H⋯*A*
C7—H7⋯O2^i^	0.95	2.46	3.404 (2)	171
C12*A*—H12*D*⋯O5^ii^	0.99	2.73	3.477 (14)	132
C17*A*—H17*C*⋯O1^iii^	0.99	2.73	3.377 (17)	124
C22—H22⋯O3^iv^	0.95	2.42	3.342 (3)	164

Symmetry codes: (i) 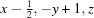; (ii) 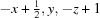; (iii) 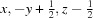; (iv) 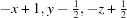.

**Table 2:** Comparison of the selected (X-ray and DFT) geometric data (Å, °)

Bonds/angles	X-ray	B3LYP/6-311G(d,p)
O1—C1	1.226 (2)	1.23817
O2—C14	1.225 (2)	1.23404
O3—C16	1.199 (2)	1.20354
O4—C16	1.324 (2)	1.36931
O4—C17	1.487 (4)	1.48849
O5—C11	1.193 (3)	1.22578
O6—C11	1.317 (3)	1.38125
O6—C12	1.476 (4)	1.47909
N1—C1	1.381 (2)	1.41268
N1—C9	1.390 (3)	1.40270
N1—C10	1.457 (2)	1.45920
N2—C14	1.349 (2)	1.38292
N2—C15	1.455 (2)	1.46732
N2—C19	1.437 (2)	1.43915
C16—O4—C17	115.0 (3)	117.00006
C11—O6—C12	115.0 (2)	117.92667
C1—N1—C9	123.54 (14)	123.13299
C1—N1—C10	116.70 (15)	115.18860
C9—N1—C10	119.72 (15)	120.66132
C14—N2—C15	116.96 (15)	115.85567
C14—N2—C19	124.11 (13)	125.08748
C19—N2—C15	117.61 (14)	119.01375
O1—C1—N1	121.49 (17)	120.57635
O1—C1—C2	123.02 (17)	123.38727
N1—C1—C2	115.48 (16)	116.01507

**Table 3:** Calculated energies

Mol­ecular Energy (a.u.) (eV)	Compound (I)
Total Energy *TE* (eV)	−40528.2845
*E*_HOMO_ (eV)	−6.1141
*E*_LUMO_ (eV)	−1.9050
Gap Δ*E* (eV)	4.2091
Dipole moment μ (Debye)	7.7590
Ionization potential *I* (eV)	6.1141
Electron affinity *A*	1.9050
Electro negativity χ	4.0095
Hardness η	2.1046
Electrophilicity index ω	3.8194
Softness σ	0.4752
Fraction of electron transferred Δ*N*	0.7105

**Table 4:** Experimental details

Crystal data	
Chemical formula	C_24_H_24_N_2_O_6_
*M*_r_	436.45
Crystal system, space group	Monoclinic, *I*2/*a*
Temperature (K)	173
*a*, *b*, *c* (Å)	16.9368 (5), 15.4130 (4), 18.4562 (6)
β (°)	109.254 (4)
*V* (Å^3^)	4548.4 (3)
*Z*	8
Radiation type	Cu *K*α
μ (mm^−1^)	0.76
Crystal size (mm)	0.32 × 0.22 × 0.14

Data collection	
Diffractometer	Rigaku Oxford Diffraction Eos Gemini
Absorption correction	Multi-scan (*CrysAlis PRO*; Rigaku OD, 2015 ▸)
*T*_min_, *T*_max_	0.710, 1.000
No. of measured, independent and observed [*I* > 2σ(*I*)] reflections	8786, 4337, 3273
*R*_int_	0.018
(sin θ/λ)_max_ (Å^−1^)	0.613

Refinement	
*R*[*F* ^2^ > 2σ(*F* ^2^)], *wR*(*F* ^2^), *S*	0.045, 0.144, 1.05
No. of reflections	4337
No. of parameters	327
No. of restraints	92
H-atom treatment	H-atom parameters constrained
Δρ_max_, Δρ_min_ (e Å^−3^)	0.22, −0.13

Computer programs: *CrysAlis PRO* (Rigaku OD, 2015 ▸), *SHELXT* (Sheldrick, 2015*b* ▸), *SHELXL2018* (Sheldrick, 2015*a* ▸) and *OLEX2* (Dolomanov *et al.*, 2009 ▸).

## supplementary crystallographic information

### Crystal data

**Table:**

C_24_H_24_N_2_O_6_	*F*(000) = 1840
*M**_r_* = 436.45	*D*_x_ = 1.275 Mg m^−^^3^
Monoclinic, *I*2/*a*	Cu *K*α radiation, λ = 1.54184 Å
*a* = 16.9368 (5) Å	Cell parameters from 3291 reflections
*b* = 15.4130 (4) Å	θ = 4.0–71.1°
*c* = 18.4562 (6) Å	µ = 0.76 mm^−^^1^
β = 109.254 (4)°	*T* = 173 K
*V* = 4548.4 (3) Å^3^	Prism, colourless
*Z* = 8	0.32 × 0.22 × 0.14 mm

### Data collection

**Table:**

Rigaku Oxford Diffraction Eos Gemini diffractometer	3273 reflections with *I* > 2σ(*I*)
Detector resolution: 16.0416 pixels mm^-1^	*R*_int_ = 0.018
ω scans	θ_max_ = 71.0°, θ_min_ = 3.8°
Absorption correction: multi-scan (CrysAlis PRO; Rigaku OD, 2015)	*h* = −20→20
*T*_min_ = 0.710, *T*_max_ = 1.000	*k* = −16→18
8786 measured reflections	*l* = −22→14
4337 independent reflections	

### Refinement

**Table:**

Refinement on *F*^2^	92 restraints
Least-squares matrix: full	Hydrogen site location: inferred from neighbouring sites
*R*[*F*^2^ > 2σ(*F*^2^)] = 0.045	H-atom parameters constrained
*wR*(*F*^2^) = 0.144	*w* = 1/[σ^2^(*F*_o_^2^) + (0.073*P*)^2^ + 1.2218*P*] where *P* = (*F*_o_^2^ + 2*F*_c_^2^)/3
*S* = 1.05	(Δ/σ)_max_ < 0.001
4337 reflections	Δρ_max_ = 0.22 e Å^−^^3^
327 parameters	Δρ_min_ = −0.13 e Å^−^^3^

### Special details

**Table:**

Geometry. All esds (except the esd in the dihedral angle between two l.s. planes) are estimated using the full covariance matrix. The cell esds are taken into account individually in the estimation of esds in distances, angles and torsion angles; correlations between esds in cell parameters are only used when they are defined by crystal symmetry. An approximate (isotropic) treatment of cell esds is used for estimating esds involving l.s. planes.

### Fractional atomic coordinates and isotropic or equivalent isotropic displacement parameters (Å^2^)

**Table:**

	*x*	*y*	*z*	*U*_iso_*/*U*_eq_	Occ. (<1)
O1	0.63298 (9)	0.28034 (12)	0.61795 (9)	0.0789 (4)	
O2	0.63302 (9)	0.47704 (9)	0.36302 (9)	0.0744 (4)	
O3	0.53817 (8)	0.38810 (10)	0.18865 (9)	0.0758 (4)	
O4	0.64912 (9)	0.42678 (11)	0.15548 (9)	0.0788 (4)	
O5	0.40565 (12)	0.20343 (12)	0.53243 (11)	0.0931 (5)	
O6	0.37031 (11)	0.24950 (13)	0.63200 (10)	0.0916 (5)	
N1	0.51128 (9)	0.34488 (10)	0.54667 (8)	0.0550 (4)	
N2	0.63939 (9)	0.33627 (9)	0.33178 (9)	0.0531 (3)	
C1	0.59207 (11)	0.31706 (13)	0.55847 (11)	0.0580 (4)	
C2	0.62452 (11)	0.33490 (12)	0.49639 (11)	0.0583 (4)	
H2	0.679690	0.316531	0.501696	0.070*	
C3	0.58017 (10)	0.37609 (11)	0.43198 (11)	0.0517 (4)	
C4	0.49587 (11)	0.40413 (11)	0.42129 (10)	0.0510 (4)	
C5	0.44640 (13)	0.44579 (14)	0.35437 (12)	0.0660 (5)	
H5	0.468954	0.457597	0.314607	0.079*	
C6	0.36578 (14)	0.46981 (16)	0.34545 (14)	0.0793 (6)	
H6	0.332464	0.498023	0.299848	0.095*	
C7	0.33343 (13)	0.45228 (17)	0.40416 (14)	0.0794 (6)	
H7	0.277547	0.468683	0.398124	0.095*	
C8	0.38016 (13)	0.41215 (14)	0.46997 (13)	0.0670 (5)	
H8	0.356741	0.401356	0.509326	0.080*	
C9	0.46250 (11)	0.38660 (11)	0.48024 (10)	0.0523 (4)	
C10	0.47616 (13)	0.32563 (14)	0.60712 (11)	0.0636 (5)	
H10A	0.448353	0.378149	0.618038	0.076*	
H10B	0.521855	0.309673	0.654605	0.076*	
C11	0.41363 (13)	0.25228 (15)	0.58447 (12)	0.0689 (5)	
C12	0.3138 (3)	0.1742 (3)	0.6220 (3)	0.1086 (14)	0.821 (8)
H12A	0.279258	0.167850	0.567335	0.130*	0.821 (8)
H12B	0.346565	0.120368	0.638828	0.130*	0.821 (8)
C12A	0.2830 (5)	0.2218 (16)	0.6170 (13)	0.112 (4)	0.179 (8)
H12C	0.247895	0.267217	0.629105	0.134*	0.179 (8)
H12D	0.256077	0.199531	0.564315	0.134*	0.179 (8)
C13	0.2606 (4)	0.1904 (5)	0.6689 (4)	0.145 (2)	0.821 (8)
H13A	0.222057	0.141575	0.663721	0.218*	0.821 (8)
H13B	0.295592	0.196529	0.722769	0.218*	0.821 (8)
H13C	0.228586	0.243799	0.651596	0.218*	0.821 (8)
C13A	0.3071 (18)	0.1534 (14)	0.6746 (15)	0.131 (6)	0.179 (8)
H13D	0.256994	0.122845	0.676250	0.197*	0.179 (8)
H13E	0.344312	0.112324	0.661214	0.197*	0.179 (8)
H13F	0.336264	0.178906	0.724946	0.197*	0.179 (8)
C14	0.61890 (11)	0.40053 (12)	0.37191 (11)	0.0545 (4)	
C15	0.67713 (11)	0.36176 (13)	0.27471 (11)	0.0575 (4)	
H15A	0.707109	0.311554	0.262692	0.069*	
H15B	0.718470	0.408417	0.295976	0.069*	
C16	0.61226 (12)	0.39333 (12)	0.20217 (11)	0.0582 (4)	
C17	0.5912 (4)	0.4604 (7)	0.0815 (3)	0.094 (2)	0.651 (18)
H17A	0.541264	0.422697	0.062247	0.113*	0.651 (18)
H17B	0.572714	0.519989	0.087923	0.113*	0.651 (18)
C17A	0.5987 (10)	0.4317 (12)	0.0724 (4)	0.111 (4)	0.349 (18)
H17C	0.603984	0.376991	0.046247	0.133*	0.349 (18)
H17D	0.538974	0.440902	0.066041	0.133*	0.349 (18)
C18	0.6387 (5)	0.4598 (10)	0.0289 (4)	0.130 (3)	0.651 (18)
H18A	0.603636	0.481446	−0.021369	0.195*	0.651 (18)
H18B	0.687974	0.497156	0.048961	0.195*	0.651 (18)
H18C	0.656703	0.400418	0.023432	0.195*	0.651 (18)
C18A	0.6304 (11)	0.5040 (12)	0.0395 (11)	0.125 (5)	0.349 (18)
H18D	0.598396	0.508837	−0.015243	0.188*	0.349 (18)
H18E	0.624789	0.557798	0.065709	0.188*	0.349 (18)
H18F	0.689542	0.494139	0.045993	0.188*	0.349 (18)
C19	0.61117 (11)	0.24813 (11)	0.33022 (10)	0.0519 (4)	
C20	0.52672 (12)	0.22939 (14)	0.30965 (11)	0.0617 (5)	
H20	0.486416	0.274673	0.297629	0.074*	
C21	0.50194 (15)	0.14319 (16)	0.30689 (13)	0.0759 (6)	
H21	0.444329	0.129315	0.294403	0.091*	
C22	0.56040 (18)	0.07794 (15)	0.32215 (14)	0.0821 (7)	
H22	0.543027	0.019086	0.319532	0.099*	
C23	0.64356 (17)	0.09746 (15)	0.34110 (15)	0.0804 (6)	
H23	0.683586	0.051977	0.351179	0.096*	
C24	0.66976 (13)	0.18267 (13)	0.34571 (12)	0.0646 (5)	
H24	0.727594	0.196038	0.359409	0.078*	

### Atomic displacement parameters (Å^2^)

**Table:**

	*U*^11^	*U*^22^	*U*^33^	*U*^12^	*U*^13^	*U*^23^
O1	0.0656 (8)	0.1041 (12)	0.0636 (9)	0.0104 (8)	0.0167 (7)	0.0190 (8)
O2	0.0863 (9)	0.0559 (8)	0.0981 (11)	−0.0102 (7)	0.0532 (9)	−0.0039 (7)
O3	0.0554 (7)	0.0897 (10)	0.0856 (10)	0.0103 (7)	0.0275 (7)	0.0135 (8)
O4	0.0712 (8)	0.1044 (12)	0.0674 (9)	0.0048 (8)	0.0319 (7)	0.0265 (8)
O5	0.1114 (13)	0.0924 (12)	0.0826 (11)	−0.0233 (10)	0.0414 (10)	−0.0174 (9)
O6	0.0886 (11)	0.1127 (14)	0.0891 (11)	−0.0207 (9)	0.0504 (9)	−0.0027 (9)
N1	0.0547 (8)	0.0648 (9)	0.0493 (8)	0.0017 (6)	0.0225 (6)	0.0000 (6)
N2	0.0529 (7)	0.0578 (8)	0.0575 (8)	−0.0016 (6)	0.0303 (7)	−0.0010 (6)
C1	0.0527 (9)	0.0646 (11)	0.0553 (10)	0.0013 (8)	0.0159 (8)	0.0006 (8)
C2	0.0471 (8)	0.0679 (11)	0.0630 (11)	0.0021 (8)	0.0225 (8)	−0.0019 (9)
C3	0.0526 (9)	0.0508 (9)	0.0580 (10)	−0.0032 (7)	0.0266 (8)	−0.0077 (8)
C4	0.0548 (9)	0.0502 (9)	0.0524 (9)	0.0023 (7)	0.0235 (8)	−0.0030 (7)
C5	0.0723 (12)	0.0695 (12)	0.0630 (11)	0.0116 (9)	0.0316 (10)	0.0067 (9)
C6	0.0751 (13)	0.0879 (15)	0.0748 (14)	0.0290 (11)	0.0247 (11)	0.0173 (12)
C7	0.0610 (11)	0.0917 (15)	0.0899 (16)	0.0266 (11)	0.0308 (11)	0.0116 (13)
C8	0.0616 (10)	0.0757 (12)	0.0731 (13)	0.0137 (9)	0.0351 (10)	0.0045 (10)
C9	0.0528 (9)	0.0532 (9)	0.0553 (10)	0.0041 (7)	0.0236 (8)	−0.0056 (7)
C10	0.0652 (11)	0.0798 (13)	0.0515 (10)	−0.0011 (9)	0.0270 (9)	−0.0004 (9)
C11	0.0698 (12)	0.0798 (13)	0.0614 (12)	−0.0012 (10)	0.0276 (10)	0.0072 (10)
C12	0.106 (3)	0.118 (3)	0.119 (3)	−0.036 (2)	0.061 (2)	−0.002 (3)
C12A	0.104 (6)	0.121 (6)	0.119 (6)	−0.018 (6)	0.048 (6)	−0.003 (6)
C13	0.118 (4)	0.180 (5)	0.169 (4)	−0.032 (3)	0.091 (3)	0.006 (4)
C13A	0.125 (10)	0.128 (10)	0.143 (10)	−0.021 (9)	0.047 (10)	−0.008 (10)
C14	0.0524 (9)	0.0545 (10)	0.0627 (11)	−0.0039 (7)	0.0273 (8)	−0.0025 (8)
C15	0.0525 (9)	0.0673 (11)	0.0622 (11)	−0.0001 (8)	0.0318 (8)	0.0034 (8)
C16	0.0606 (10)	0.0575 (10)	0.0649 (11)	0.0047 (8)	0.0322 (9)	0.0035 (8)
C17	0.092 (3)	0.129 (5)	0.068 (2)	0.034 (3)	0.035 (2)	0.039 (3)
C17A	0.084 (5)	0.137 (8)	0.107 (6)	0.019 (6)	0.027 (5)	0.048 (5)
C18	0.140 (4)	0.196 (8)	0.059 (3)	0.047 (5)	0.040 (3)	0.028 (4)
C18A	0.120 (8)	0.127 (8)	0.115 (8)	−0.022 (7)	0.021 (6)	0.048 (7)
C19	0.0596 (9)	0.0555 (9)	0.0475 (9)	−0.0043 (7)	0.0272 (8)	−0.0046 (7)
C20	0.0585 (10)	0.0710 (11)	0.0606 (11)	−0.0066 (9)	0.0262 (9)	−0.0038 (9)
C21	0.0758 (13)	0.0865 (15)	0.0706 (13)	−0.0277 (11)	0.0313 (11)	−0.0120 (11)
C22	0.1129 (19)	0.0618 (12)	0.0811 (15)	−0.0200 (12)	0.0448 (14)	−0.0102 (11)
C23	0.0962 (16)	0.0603 (12)	0.0931 (17)	0.0033 (11)	0.0428 (14)	−0.0023 (11)
C24	0.0671 (11)	0.0632 (11)	0.0704 (12)	0.0029 (9)	0.0318 (10)	−0.0031 (9)

### Geometric parameters (Å, º)

**Table:**

O1—C1	1.226 (2)	C12—C13	1.462 (5)
O2—C14	1.225 (2)	C12A—H12C	0.9900
O3—C16	1.199 (2)	C12A—H12D	0.9900
O4—C16	1.324 (2)	C12A—C13A	1.456 (6)
O4—C17	1.487 (4)	C13—H13A	0.9800
O4—C17A	1.491 (5)	C13—H13B	0.9800
O5—C11	1.193 (3)	C13—H13C	0.9800
O6—C11	1.317 (2)	C13A—H13D	0.9800
O6—C12	1.476 (4)	C13A—H13E	0.9800
O6—C12A	1.475 (6)	C13A—H13F	0.9800
N1—C1	1.381 (2)	C15—H15A	0.9900
N1—C9	1.390 (2)	C15—H15B	0.9900
N1—C10	1.457 (2)	C15—C16	1.505 (3)
N2—C14	1.349 (2)	C17—H17A	0.9900
N2—C15	1.455 (2)	C17—H17B	0.9900
N2—C19	1.437 (2)	C17—C18	1.453 (5)
C1—C2	1.451 (3)	C17A—H17C	0.9900
C2—H2	0.9500	C17A—H17D	0.9900
C2—C3	1.338 (3)	C17A—C18A	1.454 (6)
C3—C4	1.442 (2)	C18—H18A	0.9800
C3—C14	1.510 (2)	C18—H18B	0.9800
C4—C5	1.399 (3)	C18—H18C	0.9800
C4—C9	1.408 (2)	C18A—H18D	0.9800
C5—H5	0.9500	C18A—H18E	0.9800
C5—C6	1.371 (3)	C18A—H18F	0.9800
C6—H6	0.9500	C19—C20	1.384 (2)
C6—C7	1.393 (3)	C19—C24	1.378 (3)
C7—H7	0.9500	C20—H20	0.9500
C7—C8	1.360 (3)	C20—C21	1.389 (3)
C8—H8	0.9500	C21—H21	0.9500
C8—C9	1.401 (2)	C21—C22	1.374 (4)
C10—H10A	0.9900	C22—H22	0.9500
C10—H10B	0.9900	C22—C23	1.368 (4)
C10—C11	1.511 (3)	C23—H23	0.9500
C12—H12A	0.9900	C23—C24	1.380 (3)
C12—H12B	0.9900	C24—H24	0.9500
			
C16—O4—C17	115.0 (3)	H13A—C13—H13C	109.5
C16—O4—C17A	117.2 (6)	H13B—C13—H13C	109.5
C11—O6—C12	115.0 (2)	C12A—C13A—H13D	109.5
C11—O6—C12A	129.0 (10)	C12A—C13A—H13E	109.5
C1—N1—C9	123.54 (14)	C12A—C13A—H13F	109.5
C1—N1—C10	116.70 (15)	H13D—C13A—H13E	109.5
C9—N1—C10	119.72 (15)	H13D—C13A—H13F	109.5
C14—N2—C15	116.96 (15)	H13E—C13A—H13F	109.5
C14—N2—C19	124.11 (13)	O2—C14—N2	122.43 (16)
C19—N2—C15	117.61 (14)	O2—C14—C3	119.32 (16)
O1—C1—N1	121.49 (17)	N2—C14—C3	118.21 (15)
O1—C1—C2	123.02 (17)	N2—C15—H15A	109.3
N1—C1—C2	115.48 (16)	N2—C15—H15B	109.3
C1—C2—H2	118.5	N2—C15—C16	111.42 (14)
C3—C2—C1	122.92 (15)	H15A—C15—H15B	108.0
C3—C2—H2	118.5	C16—C15—H15A	109.3
C2—C3—C4	120.27 (15)	C16—C15—H15B	109.3
C2—C3—C14	121.19 (15)	O3—C16—O4	125.22 (19)
C4—C3—C14	118.37 (16)	O3—C16—C15	124.77 (17)
C5—C4—C3	122.42 (16)	O4—C16—C15	110.01 (15)
C5—C4—C9	119.62 (16)	O4—C17—H17A	110.6
C9—C4—C3	117.95 (16)	O4—C17—H17B	110.6
C4—C5—H5	119.6	H17A—C17—H17B	108.8
C6—C5—C4	120.84 (18)	C18—C17—O4	105.6 (5)
C6—C5—H5	119.6	C18—C17—H17A	110.6
C5—C6—H6	120.5	C18—C17—H17B	110.6
C5—C6—C7	119.1 (2)	O4—C17A—H17C	110.2
C7—C6—H6	120.5	O4—C17A—H17D	110.2
C6—C7—H7	119.3	H17C—C17A—H17D	108.5
C8—C7—C6	121.38 (18)	C18A—C17A—O4	107.6 (10)
C8—C7—H7	119.3	C18A—C17A—H17C	110.2
C7—C8—H8	119.7	C18A—C17A—H17D	110.2
C7—C8—C9	120.59 (18)	C17—C18—H18A	109.5
C9—C8—H8	119.7	C17—C18—H18B	109.5
N1—C9—C4	119.83 (15)	C17—C18—H18C	109.5
N1—C9—C8	121.67 (16)	H18A—C18—H18B	109.5
C8—C9—C4	118.50 (17)	H18A—C18—H18C	109.5
N1—C10—H10A	109.3	H18B—C18—H18C	109.5
N1—C10—H10B	109.3	C17A—C18A—H18D	109.5
N1—C10—C11	111.49 (16)	C17A—C18A—H18E	109.5
H10A—C10—H10B	108.0	C17A—C18A—H18F	109.5
C11—C10—H10A	109.3	H18D—C18A—H18E	109.5
C11—C10—H10B	109.3	H18D—C18A—H18F	109.5
O5—C11—O6	125.2 (2)	H18E—C18A—H18F	109.5
O5—C11—C10	125.32 (19)	C20—C19—N2	120.64 (16)
O6—C11—C10	109.49 (19)	C24—C19—N2	118.43 (16)
O6—C12—H12A	110.3	C24—C19—C20	120.85 (18)
O6—C12—H12B	110.3	C19—C20—H20	120.6
H12A—C12—H12B	108.6	C19—C20—C21	118.8 (2)
C13—C12—O6	107.0 (4)	C21—C20—H20	120.6
C13—C12—H12A	110.3	C20—C21—H21	119.9
C13—C12—H12B	110.3	C22—C21—C20	120.3 (2)
O6—C12A—H12C	113.1	C22—C21—H21	119.9
O6—C12A—H12D	113.1	C21—C22—H22	119.9
H12C—C12A—H12D	110.5	C23—C22—C21	120.2 (2)
C13A—C12A—O6	92.9 (14)	C23—C22—H22	119.9
C13A—C12A—H12C	113.1	C22—C23—H23	119.7
C13A—C12A—H12D	113.1	C22—C23—C24	120.5 (2)
C12—C13—H13A	109.5	C24—C23—H23	119.7
C12—C13—H13B	109.5	C19—C24—C23	119.3 (2)
C12—C13—H13C	109.5	C19—C24—H24	120.4
H13A—C13—H13B	109.5	C23—C24—H24	120.4
			
O1—C1—C2—C3	−179.0 (2)	C10—N1—C1—C2	178.29 (16)
N1—C1—C2—C3	0.5 (3)	C10—N1—C9—C4	−179.07 (16)
N1—C10—C11—O5	15.0 (3)	C10—N1—C9—C8	1.0 (3)
N1—C10—C11—O6	−166.24 (17)	C11—O6—C12—C13	−169.7 (5)
N2—C15—C16—O3	−8.9 (3)	C11—O6—C12A—C13A	120.5 (16)
N2—C15—C16—O4	171.66 (16)	C12—O6—C11—O5	5.8 (4)
N2—C19—C20—C21	178.30 (17)	C12—O6—C11—C10	−173.0 (3)
N2—C19—C24—C23	−177.10 (18)	C12A—O6—C11—O5	−32.5 (12)
C1—N1—C9—C4	−1.4 (3)	C12A—O6—C11—C10	148.7 (12)
C1—N1—C9—C8	178.65 (18)	C14—N2—C15—C16	−79.0 (2)
C1—N1—C10—C11	−104.9 (2)	C14—N2—C19—C20	54.7 (3)
C1—C2—C3—C4	−0.7 (3)	C14—N2—C19—C24	−128.68 (19)
C1—C2—C3—C14	174.41 (17)	C14—C3—C4—C5	6.1 (3)
C2—C3—C4—C5	−178.65 (19)	C14—C3—C4—C9	−175.41 (15)
C2—C3—C4—C9	−0.1 (3)	C15—N2—C14—O2	−2.0 (3)
C2—C3—C14—O2	−109.2 (2)	C15—N2—C14—C3	−179.57 (15)
C2—C3—C14—N2	68.5 (2)	C15—N2—C19—C20	−111.76 (18)
C3—C4—C5—C6	178.5 (2)	C15—N2—C19—C24	64.9 (2)
C3—C4—C9—N1	1.2 (2)	C16—O4—C17—C18	−157.1 (9)
C3—C4—C9—C8	−178.90 (17)	C16—O4—C17A—C18A	152.2 (17)
C4—C3—C14—O2	66.1 (2)	C17—O4—C16—O3	0.6 (5)
C4—C3—C14—N2	−116.24 (19)	C17—O4—C16—C15	−180.0 (5)
C4—C5—C6—C7	0.1 (4)	C17A—O4—C16—O3	−21.0 (10)
C5—C4—C9—N1	179.72 (17)	C17A—O4—C16—C15	158.4 (10)
C5—C4—C9—C8	−0.3 (3)	C19—N2—C14—O2	−168.50 (18)
C5—C6—C7—C8	0.2 (4)	C19—N2—C14—C3	13.9 (3)
C6—C7—C8—C9	−0.5 (4)	C19—N2—C15—C16	88.48 (19)
C7—C8—C9—N1	−179.5 (2)	C19—C20—C21—C22	−1.9 (3)
C7—C8—C9—C4	0.6 (3)	C20—C19—C24—C23	−0.5 (3)
C9—N1—C1—O1	−179.89 (19)	C20—C21—C22—C23	0.8 (4)
C9—N1—C1—C2	0.6 (3)	C21—C22—C23—C24	0.5 (4)
C9—N1—C10—C11	72.9 (2)	C22—C23—C24—C19	−0.7 (3)
C9—C4—C5—C6	0.0 (3)	C24—C19—C20—C21	1.8 (3)
C10—N1—C1—O1	−2.2 (3)		

### Hydrogen-bond geometry (Å, º)

**Table:**

*D*—H···*A*	*D*—H	H···*A*	*D*···*A*	*D*—H···*A*
C7—H7···O2^i^	0.95	2.46	3.404 (2)	171
C12*A*—H12*D*···O5^ii^	0.99	2.73	3.477 (14)	132
C17*A*—H17*C*···O1^iii^	0.99	2.73	3.377 (17)	124
C22—H22···O3^iv^	0.95	2.42	3.342 (3)	164

Symmetry codes: (i) *x*−1/2, −*y*+1, *z*; (ii) −*x*+1/2, *y*, −*z*+1; (iii) *x*, −*y*+1/2, *z*−1/2; (iv) −*x*+1, *y*−1/2, −*z*+1/2.
